# Cut to Disarm Plant Defence: A Unique Oviposition Behaviour in *Rhynchites foveipennis* (Coleoptera: Attelabidae)

**DOI:** 10.3390/insects14020200

**Published:** 2023-02-17

**Authors:** Zhi-Ying Zhang, Wei Li, Qi-Chao Huang, Liu Yang, Xiao-Lan Chen, Ru-Di Xiao, Cindy Q. Tang, Shao-Ji Hu

**Affiliations:** 1 School of Ecology and Environmental Science, Yunnan University, Kunming 650500, China; 2 School of Life Sciences, Yunnan University, Kunming 650500, China; 3 Institute of International Rivers and Eco-Security, Yunnan University, Kunming 650500, China; 4 Yunnan Key Laboratory of International Rivers and Transboundary Eco-Security, Yunnan University, Kunming 650500, China

**Keywords:** weevils, Curculionoidea, pear, callus, tannin, flavonoid, plant–insect interaction

## Abstract

**Simple Summary:**

Female *Rhynchites foveipennis* damages fruit stems when laying eggs in pears; the consequence of this interesting behaviour is poorly studied. The present study tested the hypothesis that the oviposition behaviour disarms the host plants’ defence by a series of comparative experiments. Our findings showed that when fruit stems were intact, the survival rates of eggs and larvae were only 21.3–32.6%, respectively, and the larvae weighed only 3.2–4.1 mg 30 days after oviposition. In contrast, when the fruit stems were damaged, the survival rates of eggs and larvae increased to 86.1–94.0%, respectively, and the larvae weighed 73.0–74.9 mg 30 days after oviposition. The contents of tannin and flavonoids in the pears did not change significantly, but eggs were crushed and killed by the callus in the pears. Once the stunted larvae were transferred to picked-off pears, the growth and development could recover rapidly. The authors propose that the oviposition behaviour can increase the survival and growth of the offspring, and thus is a strategy to disarm the plant defence.

**Abstract:**

Female weevils of the family Attelabidae (Coleoptera: Curculionoidea) possess a unique behaviour of partially cutting the branches connecting egg-bearing organs of their host plants during oviposition. However, the consequence of such behaviour remains unclear. Using *Rhynchites foveipennis* and its host pear (*Pyrus pyrifolia*), the present study tested the hypothesis that the oviposition behaviour could disarm the host plants’ defence. We compared the survival rates, growth rates, and performance of eggs and larvae under two conditions: (1) the fruit stems were naturally damaged by the females before and after oviposition, and (2) the fruit stems were artificially protected from the females. When fruit stems were protected from female damage, the survival rates of eggs and larvae were only 21.3–32.6%, respectively; and the larval weight was 3.2–4.1 mg 30 days after laying eggs. When the fruit stems were damaged, the survival rates of eggs and larvae reached 86.1–94.0%, respectively; and the larval weight reached 73.0–74.9 mg 30 days after laying eggs. The contents of tannin and flavonoids in the pears did not change significantly along with the oviposition and larval feeding, but weevil eggs were crushed and killed by the callus in the pears. Once the stunted larvae in branch-growing pears were moved into the picked-off ones, the growth and development recovered. The findings indicate that the oviposition behaviour can significantly increase the survival of the offspring. Our study suggested that the oviposition behaviour of attelabid weevils is a strategy to overcome plant defence.

## 1. Introduction

Plants and herbivorous insects are the most diverse groups of organisms on Earth, and their mutual relationships can be traced back hundreds of millions of years [1]. During the long-lasting course of coevolution, plants and these insects have developed a series of defence and antidefence strategies.

Plant defence mechanisms comprise constitutive and induced defence [2,3,4,5]. Constitutive defence is the built-in physical and chemical factors that prevent insect utilisation. Induced defence is the ability to produce secondary metabolites in response to a feeding/damage stimulus. In response to these plant defence systems, herbivorous insects have evolved a variety of antidefence strategies to maintain population development, including behavioural [6,7], physiological, and biochemical antidefence [8,9,10,11,12,13]. Behavioural antidefence is applied in oviposition selection, feeding avoidance, and damaging plant defence systems [6,7,14,15]. Dussourd [7] reviewed the behaviours of leaf-chewing insects that appear to function specifically to disarm host plant defences, which include vein cutting, trenching, girdling, leaf clipping, and application of fluids from exocrine glands.

The females of the weevil family Attelabidae (Coleoptera: Curculionoidea) damage or destroy the host organs before and after oviposition, such as cutting buds, shoots, petioles, leaves, and fruit branches [16,17,18], leading to the destruction of the conducting tissue. These ovipositing behaviours consume vast amounts of time and energy, although the number of eggs laid is usually limited [19,20]. The motivation for this time-consuming and laborious oviposition behaviour of attelabid weevils includes (1) induction of decay (including fungal growth), which forms preprocessing food for the larvae; and (2) providing protection by leaf rolls or other plant organs [18]. Kobayashi et al. [21] also speculated that such behaviour may prevent toxic secondary metabolites in the growing host tissue from harming the larvae. Still, no experimental studies have been reported to confirm this [22].

*Rhynchites foveipennis* Fairmare (Coleoptera: Attelabidae) is an important pest of many Rosaceae fruits across East Asia [23]. This weevil exhibits the typical oviposition behaviour of attelabid weevils. The female weevil uses its rostrum to cut the phloem near the base of the fruit stem before laying eggs. Then, it excavates an egg chamber in the fruit with its rostrum and lays an egg inside with its ovipositor. After laying the egg, the hole is plugged with faeces, leaving a blackish scar on the fruit surface. During the entire course, the male does not participate in cutting fruit stems but usually stays nearby to guard the oviposition site or to mate with the female when needed. After hatching, the larva feeds on the fruit tissue, excavates a hole in the fruit, and crawls out to pupate in the soil when reaching maturity [24].

The present study is designed to understand the oviposition behaviour of *R. foveipennis*. Experiments were conducted to compare the differences in the performance of the weevil’s offspring and the egg-bearing pear fruits. Our hypotheses are as follows: (1) the oviposition of *R. foveipennis* would trigger a series of defence reactions in the pear fruit, which would negatively affect the performance of offspring; (2) when the defence mechanism is disarmed by damaging fruit stems, the performance of offspring would improve. The findings of the present study will elucidate the consequence of the oviposition behaviour of this attelabid weevil and deepen our understanding of the insect–plant relationships in terms of basic and applied sciences.

## 2. Materials and Methods

### 2.1. Study Site and Focal Species

The study was carried out in the pear orchard (*Pyrus pyrofolia*, approximate area 0.65 hm^2^) on the Chenggong Campus of Yunnan University (24°60′ N, 102°50′ E) in Kunming, Yunnan Province (China). The pears in the orchard are 15- to 20-year-old local varieties *huangli* and *baozhuli*. The climate of the study site comprises a dry season from November to the following May and a wet season from May to October, with an annual mean temperature of 14.7 °C and an annual rainfall of approximately 900 mm.

Adult *R. foveipennis* used in the study were collected from a remotely located, pesticide-free pear orchard in Censong, Jianhe County, Guizhou Province (China) in May 2013 and 2018, respectively. *R. foveipennis* is a univoltine weevil that cannot establish continuous populations under captive rearing; thus, the present study had to collect weevils from the same pear orchard before each round of experiments in 2013 and 2018. During 2014 to 2017, unfavourable climate caused extremely low population density in Jianhe County, leading to a four-year pause of this study. Newly emerged adults were collected and transferred into captive rearing for experiments, and mature females were used in the following experiments in our laboratory at Yunnan University. The experiment was repeated in 2018 in order to compensate for problems encountered in 2013 (see Section 2.2).

### 2.2. Experimental Treatments

This experiment was designed to investigate the offspring performance of *R. foveipennis* under three types of treatments on both *baozhuli* and *huangli* varieties. Three pear fruits of similar sizes on the same branch were selected for the experiments.

Treatment A: fruit stems of pears were artificially wrapped with cotton strings to prevent the females from cutting the phloem during oviposition.

Treatment B: female *R. foveipennis* were allowed to cut the phloem of the fruit stems during oviposition, as under natural conditions.

Treatment C: protecting pear fruits from attack by *R. foveipennis* and other insects as control samples for the analysis of defensive substances only (see Section 2.3).

For treatments A and B, a female and a male *R. foveipennis* were placed with each pear fruit in a nylon-mesh bag (20 cm × 25 cm, 80 US-mesh). After oviposition, the weevils were removed, and the size of the egg-bearing fruit was measured with a Vernier calliper. Five pears from each treatment were picked immediately after the process to measure the weights and defensive substances (samples for Section 2.3) on day 0. The nylon-mesh bags were then placed back to protect the remaining pears from other insects. Pears on the same branch (with pears picked for day 0) from each treatment were sequentially collected on days 10, 20, and 30 and sent to the laboratory to measure the sizes, weights, and defensive substances (samples for Section 2.3).

Pears collected from treatments A and B were dissected to observe the survival and development of eggs and larvae under a stereomicroscope from day 10. Larvae were weighed on an electronic scale; the weights were used as indices of offspring performance. Survival rate was calculated, respectively. After observation, the dissected pears from treatments A and B were sent to the laboratory to analyse the defensive substances in comparison with pears in treatment C.

In 2013, over half of the fruits in treatment A failed because the fruit stems were only isolated by the nylon-mesh bag, and those stems were damaged again by the females during the experimental course (after oviposition). All experiments in treatment B were successful. In 2018, the protection for fruit stems was enforced by wrapping fine cotton thread before isolating the pears in nylon-mesh bags, and both treatments A and B were successful. Thus, in total, 47 fruits were used in 2013 and 142 fruits in 2018 for treatment A; 47 fruits were used in 2013 and 60 fruits in 2018 for treatment B; and 47 fruits were tested in 2013 for treatment C for analysis of defensive substances.

### 2.3. Measuring Defensive Substances

Pears from the treatments in 2013 were used in this part of analysis. Five pears collected from each treatment on days 0, 10, 20, and 30 in Section 2.2 were used to measure defensive substances after measuring the sizes, weights, and offspring performance. Kim and Lee [25] reported that phenolic compounds in apple fruits might be a defensive substance that suppressed the larval survival rate of *Carposina sasakii*. In this study, we analysed tannin and flavonoid contents to represent defensive substances in pears collected from all three treatments.

#### 2.3.1. Tannin Content

The tannin content (*y*_1_) of the treated fruits was determined by the regression equation *y*_1_ = 0.0623*x*_1_ − 0.0003 (*R*² = 0.9999), which was established using the standard tannic acid solution of 0, 1.0, 2.0, 3.0, 4.0, 5.0, and 6.0 μg/mL on a photospectrometer under 760 nm according to the method described by Julkunen-Titto [26]. In the equation, *x*_1_ is the absorbance of the fruit powder extract.

The tannin extraction from fruit powder was prepared with the following protocol: A total of 0.5 g dried pear was weighed, ground, passed through a 40-mesh screen (accuracy 0.0001 g), and added to 30 mL ethanol. The mixture was incubated at 60 °C for 30 min and then filtered. The residue was washed 2–3 times with 70% ethanol and tested with 5% FeCl_3_ solution until no blue-black or green-black colour appeared, indicating complete extraction of tannin [27]. The supernatant was collected and diluted to volume in a 100 mL volumetric flask.

Upon testing, 2 mL of fruit powder extract, 5 mL of molybdo-phosphotungstate reagent, and 10 mL of saturated sodium carbonate (Na_2_CO_3_) solution were mixed and diluted with water in a 100 mL volumetric flask. After 30 min, the absorbance (*x*_1_) was measured under 760 nm light on a photospectrometer.

#### 2.3.2. Flavonoid Content

The flavonoid content (*y*_2_) of treated fruits was determined by the regression equation *y*_2_ = 1.0937*x*_2_ + 0.0022 (*R*^2^ = 0.9998), which was established using the standard rutin solution of 0, 1.0, 2.0, 3.0, 4.0, and 5.0 mL on a photospectrometer under 510 nm according to the method described by Jia et al. [28]. In the equation, *x*_2_ is the absorbance of the fruit powder extract.

The flavonoid extraction from fruit powder was prepared with the following protocol: A total of 0.5 g of fruit powder was mixed with 70 mL of 70% ethanol in a reflux flask and extracted at 80 °C for 2 h. The supernatant was collected and diluted with 70% ethanol to volume in a 100 mL volumetric flask.

Upon testing, 1.0 mL extract was placed in a 10 mL volumetric flask and diluted to 5 mL with 30% ethanol. Then, 0.3 mL of 5% NaNO_2_ solution was added, and 0.3 mL of 10% Al(NO_3_)_3_ was added and mixed well five minutes later, then 2 mL of 1M NaOH was added six minutes later, and finally diluted to volume with 30% ethanol. The absorbance (*x*_2_) was measured under 510 nm on a photospectrometer.

### 2.4. Data Analysis

For continuous variables such as pear fruit size, larval weight, and the contents of tannin and flavonoid, average values with standard deviations were calculated and plotted using Microsoft Excel 2016. Statistical significance was tested on fruit size, larval weight, and the contents of tannin and flavonoid on days 10, 20, and 30 separately using a one-way ANOVA in SPSS 28.0 (IBM Corporations, Armonk, NY, USA).

For the mortality of eggs and the survival rate of larvae, the average values were calculated and plotted to show the trends using Microsoft Excel 2016. The statistical significance was tested using χ^2^ test in SPSS 28.0.

## 3. Results

### 3.1. Effects on Fruit Growth

**Figure 1:**
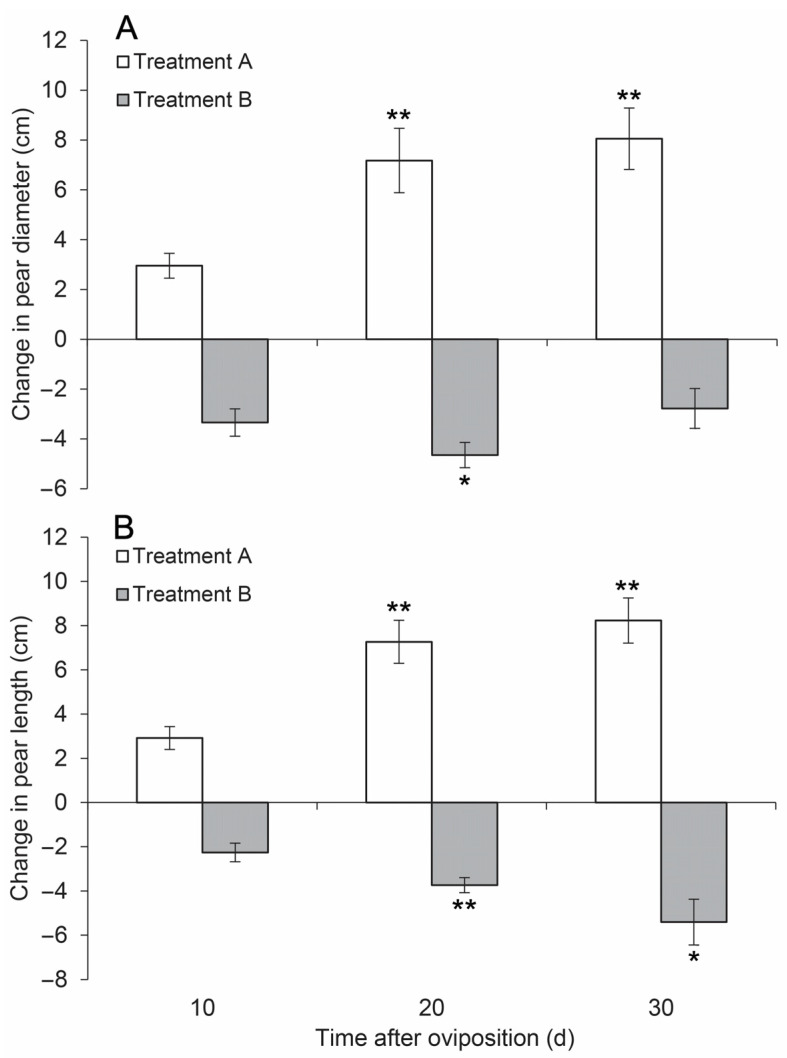
Changes in the diameter (**A**) and length (**B**) of pear fruits on 10, 20, and 30 d after oviposition by female *R. foveipennis* in 2013, Vertical bars are standard deviations; * statistical significance at 0.05 level, ** statistical significance at 0.01 level.

The diameters and lengths of the pears in treatment A increased after oviposition, and obvious increases were observed on days 10 and 20 (Figure 1). However, after day 20, the change in the fruit size was less obvious compared to the previous stages.

In contrast, the diameters and lengths of the pears in treatment B began to decrease after day 10. Some pears in treatment B even dropped to the ground on day 30 when it rained. After soaking in rainwater, the diameters of these dropped fruits increased (Figure 1).

The ANOVA analysis on diameter and lengths showed that both indices were not significantly different on day 10 from the initial stage (day 0). However, on days 20 and 30, the differences all turned significant at 0.01 level in treatment A. In treatment B, the diameter was significantly different at 0.05 level on day 20, and showed no significant difference on day 30. Similarly, the length in treatment B was significantly different at 0.01 level on day 20, but less significant (at 0.05 level) on day 30 (Figure 1).

The results clearly showed that when the fruit stems were intact (under protection against the female *R. foveipennis*), the pears could grow normally, even after oviposition (Figure 1). However, the pears gradually decreased in size after the fruit stems were damaged by the females in the natural oviposition process (Figure 1).

### 3.2. Effects on Offspring Survival Rate and Development

**Figure 2:**
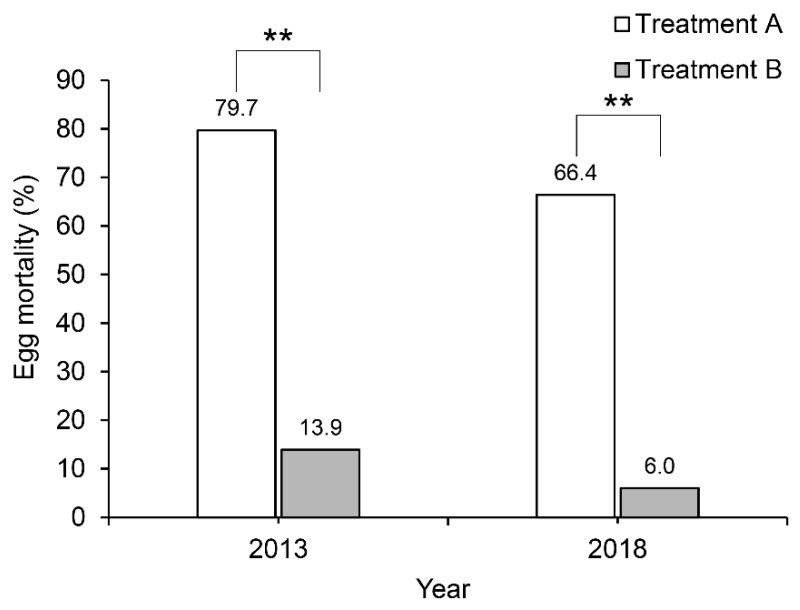
Mortality of eggs of *R. foveipennis* in pear fruits under treatments A and B in year 2013 and 2018. The mortalities in treatment A were significantly higher than those in treatment B in both years; ** statistical significance at 0.01 level.

**Figure 3:**
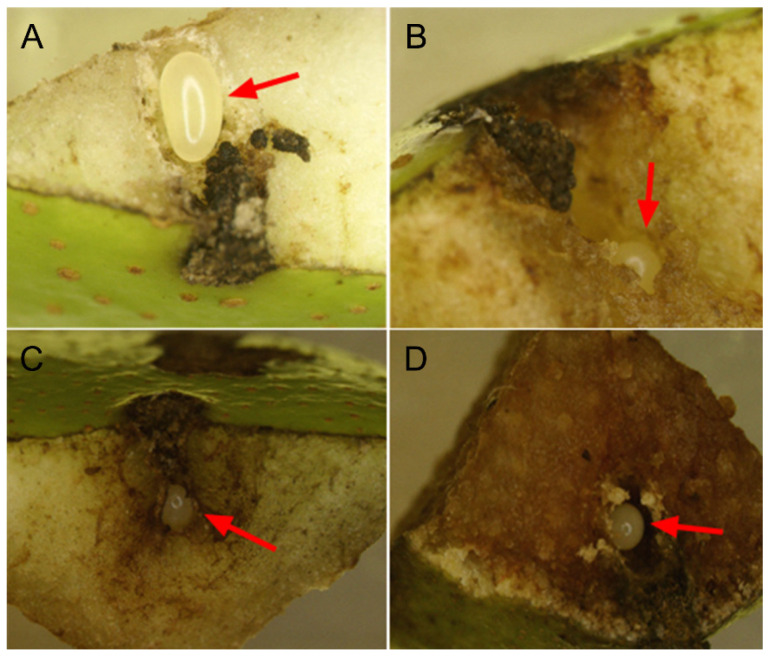
Egg chambers of *R. foveipennis* after oviposition: (**A**) egg laid after 2 days, (**B**,**C**) chamber filled with callus with crushed egg in treatment A after 5 days, (**D**) normal chamber with intact egg in treatment B after 5 days. Red arrow in each photo indicates the position of an egg.

In 2013, the mortality rates of eggs on day 10 were 79.7% in treatment A (*n* = 79) and 13.9% in treatment B (*n* = 144) (Figure 2). In 2018, the mortality rates were 66.4% in treatment A (*n* = 146) and 6.0% in treatment B (*n* = 100) (Figure 2). In pears with failed eggs, the egg chambers were severely occupied by the fruits’ secondary growing tissues (callus). All failed eggs were crushed to a shrivelled size and damaged significantly (Figure 3).

**Figure 4:**
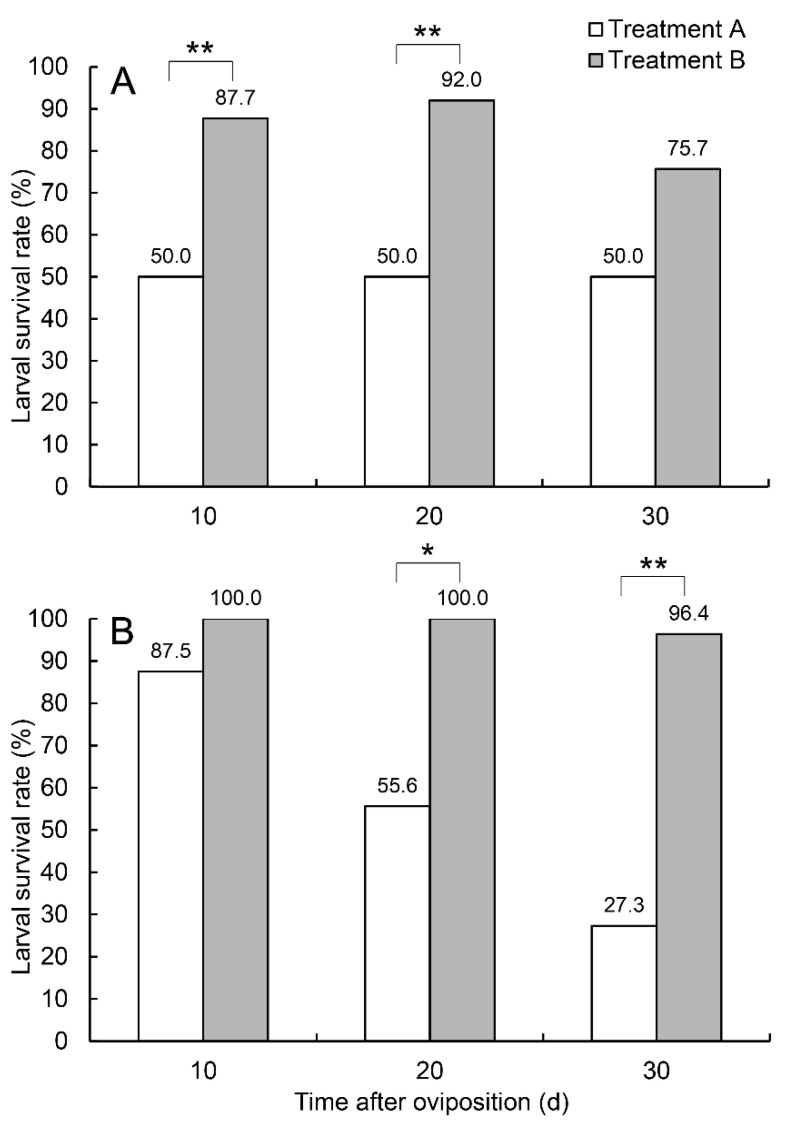
Larval survival rates of *R. foveipennis* on days 10, 20, and 30 after oviposition under treatments A and B in 2013 (**A**) and 2018 (**B**); * statistical significance at 0.05 level, ** statistical significance at 0.01 level.

The larval survival rates on days 10, 20, and 30 in treatment A were 50% (*n* = 8), 50% (*n* = 4), and 50% (*n* = 4), in 2013, respectively, and 87.5% (*n* = 32), 55.6% (*n* = 18), and 27.3% (*n* = 11) in 2018. The larval survival rates on days 10, 20, and 30 in treatment B were 87.7% (*n* = 57), 92.0% (*n* = 50), and 75.7% (*n* = 37) in 2013, and 100% (*n* = 22), 100% (*n* = 10), and 96.4% (*n* = 28) in 2018 (Figure 4).

**Figure 5:**
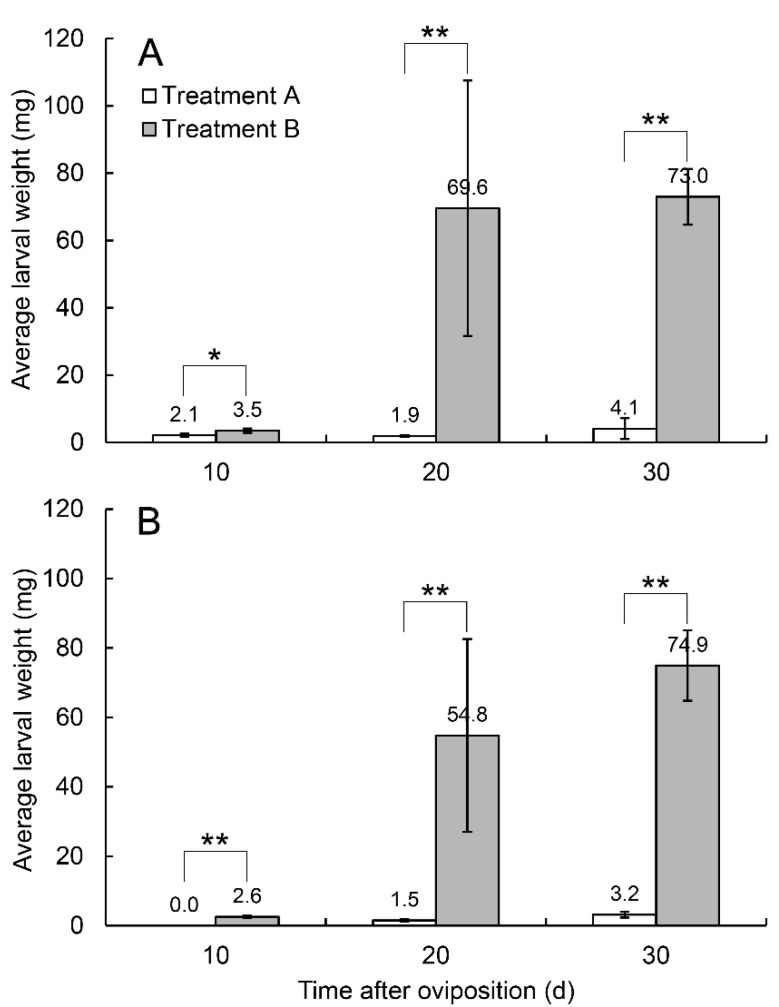
Average weights of larvae of *R. foveipennis* on days 10, 20, and 30 after oviposition under treatments A and B in 2013 (**A**) and 2018 (**B**). Vertical bars are standard deviations; * statistical significance at 0.05 level, ** statistical significance at 0.01 level.

In 2013, the average weight of the same batch of larvae only reached 4.1 mg in treatment A on day 30, while in treatment B, the average larval weights on days 10, 20, and 30 were 3.5 mg (*n* = 50), 69.6 mg (*n* = 46), and 73.0 mg (*n* = 28) (Figure 5). In 2018, the average weight of larvae only reached 3.2 mg (*n* = 9) in treatment A but reached 74.9 mg (*n* = 27) in treatment B on day 30 (Figure 5). The larvae in treatment A failed to develop and stopped at the first instar, while the larvae in treatment B developed normally, with their weights increasing with time; some larvae even reached maturity and emerged from the pears searching for pupating sites.

### 3.3. Changes in Defence Substances

**Figure 6:**
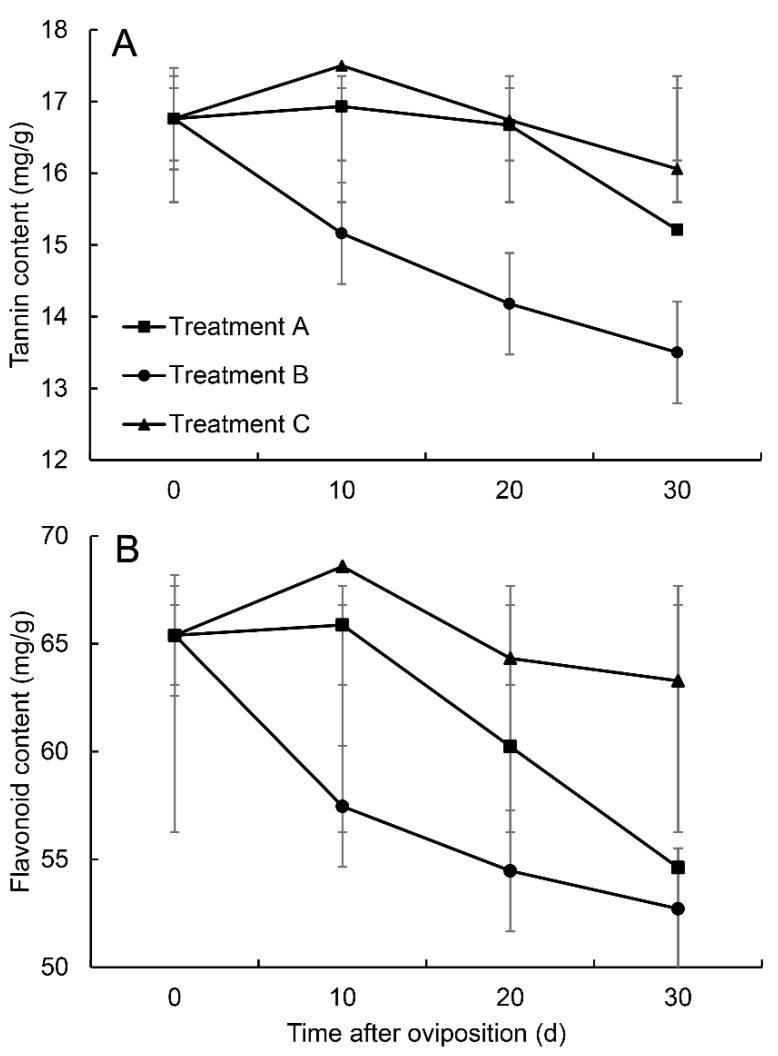
Change in defence substances after oviposition of *R. foveipennis*: (**A**) change in tannin content; (**B**) change of flavonoid content on days 10, 20, and 30 in treatments A, B, and C in 2013. Y-axes do not start from zero. Five (pear) replicates from each treatment at each time; vertical bars are standard deviations.

The tannin content in all pears from three treatments increased on day 10 but decreased with time afterwards (Figure 6). The tannin content in the pears of treatment A showed no significant difference from that in treatment C on days 10, 20, and 30, while it was significantly different between treatment B and both of treatments A and C (Table 1). Although the tannin content in treatment A was very similar to that in treatment C on day 20, the level was still lower compared to that in treatment C on day 30. On the contrary, the tannin content in the pears of treatment B decreased more quickly with time and was significantly lower on days 10, 20, and 30 after oviposition than that in treatments A and C (Figure 6).

Similarly, the flavonoid content in the pears in all treatments increased on day 10 but decreased afterwards (Figure 6). However, the flavonoid contents in the pears of treatments A, B, and C all showed significant differences from each other on days 10, 20, and 30 (Table 1). The decreasing trends in the pears of treatments A and C were similar, while that in treatment B decreased more quickly, especially during the first 10 days.

## 4. Discussion

Our experiment showed that the sizes of pear fruits gradually decreased after natural oviposition in treatment B (Figure 1), since the females of *R. foveipennis* must damage or destroy the conducting tissues of the egg-bearing organs, like most species of the family Attelabidae [16,17,18]. Although being still ‘connected’ on the branches, the egg-bearing pears stopped growing and withered after a short period of time. Female *R. foveipennis* are particularly persistent in destroying the stems of suitable fruits before oviposition once they touched the stems in our experiments. Females tried every possible way to damage the fruit stems even when they were protected inside nylon-mesh bags, such as those failed samples in treatment A in 2013. However, when this behaviour was completely prohibited, the females also laid eggs in suitable pears without damaging the fruit stems. The findings in this study and our other unpublished studies involving *Mechoris ursulus* and *M. cumulates* both suggest that the offspring of the fruit-boring weevils could not complete their development if the eggs were laid in living branch-growing fruits (unpublished data). Hence, stem-damaging behaviour is crucial to the survival and reproduction of Attelabidae, which can be interpreted as an evolutionary ’arms race’ to interact with host plants. The 2000-plus known species of Attelabidae [29] contain diverse feeding modes, including those feeding on living plant tissues, inside living tissues, and on dead/decayed tissues [18]. In Curculionoidea, the ancestral groups usually only feed on dead/decayed plant tissues, while the advanced (evolved) groups feed on/inside living plant tissues [30,31]. Such diverse feeding modes along with the unique ovipositing behaviour of Attelabidae may play a bridging role in the phylogenetic evolution of Curculionoidea. Many herbivorous insects cut off plant parts to consume to avoid plant-induced defences [32,33]. However, these behaviours are initiated by larvae themselves, instead of adults preparing suitable development condition for offspring, such as female *R. foveipennis*.

When female *R. foveipennis* were prevented from damaging fruit stems, more than 70% of eggs died, while others failed to develop normally. Similar results were also obtained in our experiments with *M. ursulus*, which partially cuts the fruit branches of Fagaceae plants [20]. Eggs are the initial and most vulnerable stage in the life cycle of insects; therefore, most herbivorous insects lay their eggs on the exterior parts of host plants, and the hatched larvae start feeding on the same parts of host plants, e.g., butterflies and leaf beetles. Some insects, such as fruitworm moths (Lepidoptera: Carposinidae), lay their eggs on the exterior parts of the host plant even though the larvae bore into the fruits after hatching. The mortality of eggs laid on the exterior parts of host plants are commonly higher due to dehydration, parasitism, and predation. During the course of evolution, most Curculionoidea species developed a rostrum to feed deeply and to facilitate oviposition by excavating chambers in the host tissues in order to lay eggs inside, which provide eggs with more physical protection and enable the larvae to feed on fresh tissues after hatching [34,35]. However, such innovation inevitably faces the risk of induced defence from host plants. Our study first demonstrated that eggs in branch-growing pears were crushed to death by the callus produced by the sarcocarp (Figure 3), and then detected the oviposition-induced increase in defensive substances (Figure 5; Table 1). In our unpublished study, the egg survival rate of *M. ursulus* also increased when the egg-bearing acorns were cut off (Zhang et al., unpublished data). However, this phenomenon is rarely reported by other available research [36,37]. Further studies need to verify whether this behaviour is common in the family Attelabidae, except for the cradle-making species that lay eggs on the exterior parts of their host plants, representing the evolutionarily advanced group of the family Attelabidae [21].

The hatched larvae of *R. foveipennis* in branch-growing pears failed to develop normally and remained in the first instar in our experiments. Larval mortality in treatment A was over 20-fold compared to that in treatment B (Figure 2). Similar phenomena were also reported for the fruitworm moth *Carposina sasakii* [25]. Ishiguri and Toyoshima [38] discovered that the larvae of *C. sasakii* had a higher survival rate and a shorter period of development in picked-off apples than in branch-growing ones. Our findings on the contents of defensive substances demonstrated a sharper decrease in both tannin and flavonoid in treatment B, in which the fruit stems were damaged by the female *R. foveipennis* (Figure 6; Table 1). It must be noted that the tannin and flavonoid contents in treatment A also decrease more than those in treatment C, although no statistical significance was detected (Figure 6; Table 1). It is generally accepted that tannin is toxic to most insect herbivores [39], and very recent research using artificial diet containing different levels of tannic acid demonstrated that at low concentrations, tannin can promote feeding and food utilisation in *Hyphantria cunea* larvae, while at high concentrations, inhibitory effects were observed [40]. Moreover, the present study also discovered that the larval development of *R. foveipennis* can resume to near normal after being transferred from branch-growing pears to picked-off ones (see Supplementary Materials). Therefore, the authors believed that the host plant’s defence system was triggered by both oviposition and cutting caused by female *R. foveipennis* and the feeding larvae; in the meantime, female cutting must have had the most powerful outcome. The higher level of defence substances might hinder larval growth, despite the possibility that weevils may have been adapted to high tannin content, as in the cases reported for *Curculio arakawai* and *C. dentipes* [41].

## 5. Conclusions and Limitations

The present study confirmed that the offspring survival rate and performance would be reduced when the stem-damaging behaviour of the female *R. foveipennis* is prohibited. Protected egg-bearing pears could produce a callus to crush eggs and kill them first, and the larvae left in such pears would suffer from higher contents of tannin and flavonoid compared to the ones under natural conditions, in which the fruit stems were damaged by female *R. foveipennis*. When fruit stems were damaged, the tannin and flavonoid contents could decrease more quickly on the first 10 days, leading to an increased survival rate and better development of larvae in the pears. Thus, the authors speculated that tannin and flavonoid may play the role of protease inhibitor, which could halt the growth of larvae.

Due to the difficulty in rearing *R. foveipennis* under captive conditions, some experiments (e.g., the survival rate of larva) in the present study could only be performed with a limited number of samples, which may introduce bias. Future research must test these conclusions before using them as a guide. The present study did not test whether significant change existed in pear fruit size in treatment C; thus, the growing plus oviposited effects in treatment A must also be accepted cautiously.

## Figures and Tables

**Table 1:** The ANOVA analysis for content differences of tannin and flavonoid on days 10, 20, and 30 in treatments A, B, and C in 2013.

Data Pair	Substance	Day 10			Day 20			Day 30		
		Sum of Squares	*F*	*P*	Sum of Squares	*F*	*P*	Sum of Squares	*F*	*P*
A-B	tannin	0.0006	612.3235	<0.001 **	0.4673	312.381	<0.001 **	0.1902	85.9985	<0.001 **
	flavonoid	0.3000	162.7493	<0.001 **	0.0002	95.5981	<0.001 **	<0.0001	9.6131	0.0048 **
B-C	tannin	0.0011	60.1820	<0.001 **	0.4916	207.4074	<0.001 **	0.4261	30.9729	<0.001 **
	flavonoid	0.5207	30.4147	<0.001 **	0.0007	66.2448	<0.001 **	0.0007	27.8848	<0.001 **
C-A	tannin	0.0001	3.4896	0.0699	0.0003	0.1184	0.7332	0.0468	3.3359	0.0802
	flavonoid	0.0302	1.7141	0.1987	0.0001	13.5022	0.0010 **	0.0005	17.5212	<0.001 **

** Significance at 0.01 level.

## Data Availability

The data presented in this study are available on request from the corresponding authors. The data are not publicly available due to being a subset of an ongoing research project.

## Supplementary Materials

The following supporting information can be downloaded at https://www.mdpi.com/article/10.3390/insects14020200/s1: Figure S1: Comparison of the stunted and recovered larvae of *R. foveipennis*: A_1_: stunted larva in branch-growing pear on June 30, B: three normally developing larvae in picked-off pear on June 30, A_2_: recovered larva of A_1_ in picked-off pear on July 10.
