# Supplementary material for: Cut to Disarm Plant Defence: A Unique Oviposition Behaviour in Rhynchites foveipennis (Coleoptera: Attelabidae)
Source: Insects. 2023 Feb 17;14(2):200. doi: 10.3390/insects14020200 (PMC9965434; doi:10.3390/insects14020200)
Supplement: Supplementary file 1 [file insects-14-00200-s001.zip › insects-2200188-supplementary.pdf]

Supplementary material

* The following experiment was performed to test if larvae in the branch growing pears can resume growth when artificially moved into picked-off pears. Due to limited experiment outcomes, this part is not included in the main text.

Development recovery of stunted larvae

On day 20 after treatments, pears were dissected to collect the stunted larvae from pears in treatment A. Larvae in treatment B nearly reached maturity and thus were not used for this experiment. Dying larvae were excluded, the remaining actively living but stunted larvae (n = 6) were then placed into picked-off pears using the artificial inoculation technique. Selected pears were sterilised with 75% ethanol solution, then a 3-mm hole was drilled into the pear with a sterilised awl to simulate the ovipositor insertion of female *R. foveipennis*. The purpose of this experiment was to determine whether eliminating host defence would resume the development of larvae.

On day 10 after inoculation, all picked-off pears with larvae were dissected to evaluate larval performance. Once the evaluation was finished, all larvae were again placed into the next group of picked-off pears to continue the experiment. Six days later, another evaluation was done, and all larvae were again placed into the third group of picked-off pears. We again dissected the fruit to observe the developmental recovery of the larvae.

Artificial inoculation of larvae

This experiment was designed to investigate the difference in offspring performances of *R. foveipennis* between branch-growing pears and picked-off pears. This experiment aimed to test the hypotheses obtained from the previous experimental treatments — whether host defence can reduce offspring performance of *R. foveipennis*.

Larvae at different stages were collected from pears on which eggs were naturally laid. All collected larvae were weighed and divided into two groups, each group contained 15 larvae with similar stages and weights.

The experiment was carried out in the pear orchard. For group I, larvae were placed into 15 branch-growing pears using the artificial inoculation technique mentioned above, and protected by nylon-mesh bags. For group II, the same protocol was applied to 15 picked-off pears.

On day 12 after the experiment, all 30 pears (both branch-growing and picked-off ones) were dissected to extract the larvae. The survival rate was calculated, and the larvae in the two groups were weighed on a scale. Both indices were used for offspring performance.

Parallel experiments with the same protocol and the same number of newly hatched larvae reared in the laboratory were carried out on the *huangli* and *baozhuli* varieties. On day 15 after the experiment, we dissected all fruits to obtain the larval survival rate and weights.

Recovery of stunted larvae

On day 20 after the treatments, the larvae in treatment A almost remained at the newly hatched status, with an average weight of 1.7 mg (Figure S1). After transferring the stunted larvae into picked-off pears, the development gradually resumed, although the development rate was still lower after another 10 days compared to the larvae living in picked-off pears from the beginning (Figure S1). After another 6 days, the development rate of these stunted larvae increased more rapidly (Figure S1).

Effects on artificially inoculated larvae

The development of larvae in the branch-growing pears in group I was hindered, some larvae even died in the pears, and only a limited number of larvae continued to feed and entered the endocarp to consume seeds. The average weight of the larvae in group I only increased by 7.8 mg compared to the original status.

However, in the picked-off pears in group II, all larvae developed normally and consumed the sarcocarp. The average weight of the larvae in group II increased by 48.1 mg compared to their original status. Some larvae even reached maturity and emerged from the pears searching for pupating sites.

On day 15 after the parallel experiments with *huangli* and *baozhuli* varieties, the pears were dissected to examine the larval performance. The average weight of larvae in group I was 3.3 mg in *huangli* variety and 2.3 mg in *baozhuli* variety. In comparison, the average weight of larvae in group II was 52.4 mg and 37.9 mg, respectively.

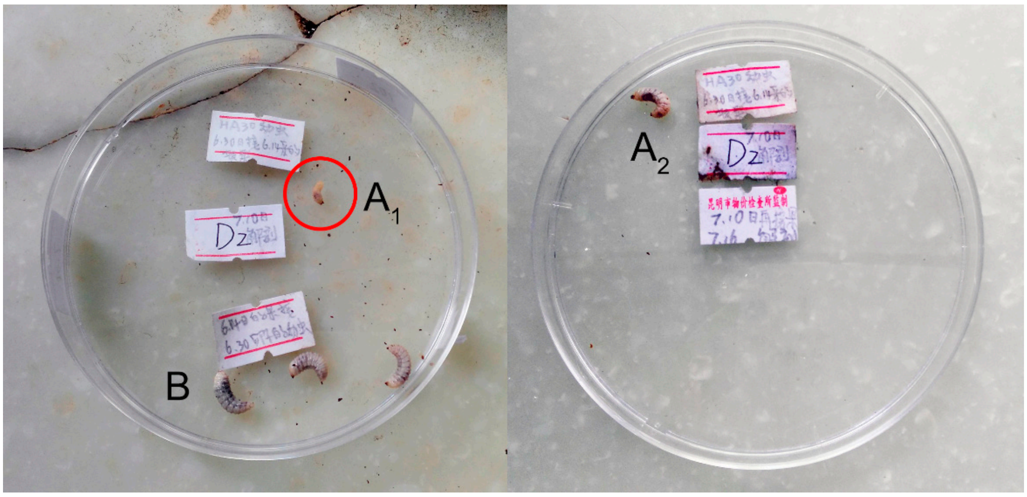

Figure S1. Comparison of the stunted and recovered larvae of *R. foveipennis*: A_1: stunted larva in branch-growing pear on June 30, B: three normally developing larvae in picked-off pear on June 30, A_2: recovered larva of A_1 in picked-off pear on July 10.
